# Automated Verification of Parallel Nested DFS

**DOI:** 10.1007/978-3-030-45190-5_14

**Published:** 2020-03-13

**Authors:** Wytse Oortwijn, Marieke Huisman, Sebastiaan J. C. Joosten, Jaco van de Pol

**Affiliations:** 8 Johannes Kepler University, Linz, Austria; 9 University of Birmingham, Birmingham, UK; 10 Department of Computer Science, ETH Zurich, Zurich, Switzerland; 11 Formal Methods and Tools, University of Twente, Enschede, The Netherlands; 12 Dartmouth College, Hanover NH, USA; 13 Department of Computer Science, Aarhus University, Aarhus, Denmark

## Abstract

Model checking algorithms are typically complex graph algorithms, whose correctness is crucial for the usability of a model checker. However, establishing the correctness of such algorithms can be challenging and is often done manually. Mechanising the verification process is crucially important, because model checking algorithms are often parallelised for efficiency reasons, which makes them even more error-prone. This paper shows how the VerCors concurrency verifier is used to mechanically verify the parallel nested depth-first search (NDFS) graph algorithm of Laarman et al. [25]. We also demonstrate how having a mechanised proof supports the easy verification of various optimisations of parallel NDFS. As far as we are aware, this is the first automated deductive verification of a multi-core model checking algorithm.

### 1 Introduction

Model checking is an automated procedure for verifying behavioural properties of reactive systems. To avoid a false sense of safety, it is essential that model checkers are themselves correct. However, model checkers use ever more ingenious algorithms [12] and even parallel implementations [2] to be able to combat the large state spaces of critical industrial systems, which makes it increasingly difficult to guarantee their correctness.

This paper focusses on the mechanical verification of a *multi-core* model checking algorithm for detecting accepting cycles in automata, called nested depth-first search (NDFS). This algorithm solves the model checking problem for Linear-time Temporal Logic (LTL), a widely used logic for specifying reactive systems. Multi-core NDFS is developed by Laarman et al. in 2011 [25] and is currently deployed in the high-performance model checker LTSmin [23].

The mechanical verification of parallel NDFS is carried out in VerCors [6], a verifier based on concurrent separation logic that targets real-world concurrent

⋆ This research has been performed while working at the University of Twente.

and parallel programs. The presented verification is inspired by a previous mechanical verification of *sequential* NDFS [37] that was carried out in Dafny [30].

This paper demonstrates the feasibility of mechanical program verification of parallel graph algorithms, like multi-core NDFS. To the best of our knowledge we present the first mechanical verification of a parallel graph algorithm. Our formalisation provides reusable components that can be used to verify variations of parallel NDFS, as well as other algorithms for parallel model checking.

Before listing our contributions (§1.3) we first provide more background on model checking algorithms (§1.1) and related work on their verification (§1.2).

#### 1.1 Background on Model Checking

Pnueli introduced the Linear-time Temporal Logic (LTL) [36] to specify properties of reactive systems. The model checking problem [12] decides whether a transition system satisfies a given LTL property. The automata-based approach [45] reduces the model checking problem to the graph-theoretic problem of checking the reachability of accepting cycles. Reachability of accepting cycles in directed graphs can be checked in linear time, with the nested depth-first search (NDFS) algorithm [13,19,41], which forms the basis of the Spin model checker [17].

Several distributed and parallel model checking algorithms have been proposed, to allocate more memory and processors to the problem [2]. NDFS is based on depth-first search, which is considered hard (impossible) to parallelise efficiently [39]. For distributed approaches, the best strategy is to turn to BFS algorithms [3], which are straightforward to parallelise but at the cost of increasing the amount of work beyond linear time. For the shared-memory setting, swarm verification was proposed [18], where each worker runs its own instance of NDFS. Various DFS-based multi-core algorithms for full LTL model checking have been devised for this strategy [14,15,25]. This paper considers the version by Laarman et al. [25], which is a parallel version of improved sequential NDFS [41].

The correctness of parallel NDFS is quite subtle. In particular, parallel DFS does not fully respect a global depth-first ordering, since each worker maintains its own search stack, yet the correctness of NDFS depends on the search order. Also, to realise speedups, the implementation avoids locking shared data structures by using atomics. This raises the question whether the implementation of a parallel model checker, meant to verify the correctness of safety-critical systems, is itself correct. For this reason the original paper [25] contains a detailed *pen-and-paper* correctness proof, which is based on a number of invariants.

#### 1.2 Related Work

To raise the level of confidence in model checkers, one approach is to certify each of their individual runs. Obviously, the counterexample returned by a model checker is itself a certificate that can easily be verified independently. However, double-checking the absence of errors is harder. Namjoshi [33] proposed to instrument a $\mu$-calculus model checker, to generate a deductive proof that can be checked independently, also in case the property holds. Recently, an IC3-style symbolic LTL model checker has been extended with deductive proofs as well [16]. However, these approaches do not prove correctness of the model checking algorithm, but only validate its outcome for each specific use.

Alternatively, one can formalise the model checking algorithm and its correctness proof in an interactive theorem prover. An early example of this approach was the verification of a model checker for the modal $\mu$-calculus in Coq [43]. A framework for verifying sequential depth-first search algorithms was developed in Isabelle [27,28], and applied to the verification of NDFS with partial order reduction [9] as well as a model checker for timed automata [47]. The recent formalisations of Tarjan's SCC algorithm [10] fit in the same line of research. These approaches require to model and verify the algorithm in an interactive theorem prover, allowing one to use the full power of the theorem prover.

If one wishes to verify the code of the algorithm directly, yet another approach is to model the algorithm and its specification in a (semi-)automated program verifier, where the code is enriched with sufficient annotations to prove its correctness. This approach was followed for several standard sequential graph algorithms in Why3 [46] and for sequential NDFS in Dafny [37]. However, there is hardly any work on automated verification of parallel graph algorithms. Raad et al. [38] verified four concurrent graph algorithms in the context of CoLoSL, but the proofs have not been automated. Sergey et al. [42] verified a concurrent spanning tree algorithm, but interactively, through an embedding in Coq.

To support the verification of shared-memory parallel software, program verifiers typically use concurrent separation logic. VeriFast [20] aims at sequential and multi-threaded C and Java programs. VerCors [6] verifies concurrent programs in Java and OpenCL, by applying a correctness-preserving translation into a sequential imperative language, delegating the generation of the verification conditions to Viper [32] and their verification ultimately to Z3 [31].

#### 1.3 Contributions and Outline

This paper discusses the mechanical verification of the *parallel* NDFS algorithm of Laarman et al. [25] using VerCors. To the best of our knowledge, this is the first mechanical verification of a parallel graph (and model checking) algorithm.

Section 2 recalls both sequential and parallel NDFS (§2.1–2.2), and gives preliminaries on concurrency verification with VerCors (§2.3). It also explains that parallel NDFS uses various colour markings on the input graph to administer the status of the nested searches of workers. Some of these colours are local to a single worker, while other colours are globally shared among all workers.

Section 3.1 presents our new (informal) correctness proof of parallel NDFS, that is based on a number of global invariants on the possible colour configurations. The main challenge lies in proving completeness, which is particularly difficult since workers can delegate the detection of accepting cycles to other workers. To be able to mechanise our completeness proof, we contribute a new invariant (Lemma 4) that guarantees the preservation of so-called *special paths*. This allows to circumvent using the complicated inductive argument used by [25].

Section 3.2 discusses how parallel NDFS is specified in VerCors. In particular, this requires the specification of permissions, to verify data race-free access to shared data structures. Moreover, we encode the colour maps and the transition relation of the input automaton as matrices, which greatly contribute to the feasibility of proof checking. We also explain how atomic updates are specified, which was left implicit in the high-level pseudo code. Similarly, we implement asymmetric termination detection: if one worker finds a counterexample, all workers can terminate immediately; if, on the other hand, all workers have completely finished their exploration, only then may one conclude that the model is correct.

Section 3.3 explains the techniques to formalise the full functional correctness proof in VerCors. In particular, this requires the distribution of permissions and invariants over threads and locks, and the introduction of auxiliary ghost state to track the precise progress of the various nested search phases of all workers.

Section 4 demonstrates how our verification is reused to verify optimisations to the algorithm. In particular, we check the optimisation "early cycle detection" that, for weak LTL properties, detects all cycles in the outer search instead of the nested inner search. We also propose and verify a repair to the "all-red" extension, by inserting an extra check that was missing in [25]. This extension improves the speedup of parallel NDFS by sharing more global information.

Finally, Section 5 concludes with a perspective on reusing our techniques for verifying other parallel graph algorithms.

### 2 Preliminaries

Section 2.1 recalls the standard sequential NDFS algorithm for finding reachable accepting cycles in automata. We verified a parallel version of NDFS, which is introduced in Section 2.2. The verification has been performed with VerCors; Section 2.3 gives prerequisites on concurrency verification and separation logic.

Before discussing the NDFS algorithms, let us first recall the basic definitions of automata and accepting cycles. An *automaton* $G$ is a quadruple $(\mathcal{S}, s_I, \mathsf{succ}, \mathcal{A})$ consisting of a finite set $\mathcal{S}$ of *states*, an *initial state* $s_I \in \mathcal{S}$, a *next-state relation* $\mathsf{succ} : \mathcal{S} \to 2^{\mathcal{S}}$ and a set $\mathcal{A} \subseteq \mathcal{S}$ of *accepting states*. A *path* in $G$ is a sequence $P = s_0, \ldots, s_{n+1}$ of $\mathcal{S}$-states so that $s_{i+1} \in \mathsf{succ}(s_i)$ for every $0 \leq i \leq n$. The notation $|P| \triangleq n + 2$ denotes the *length of* $P$, $P[i] \triangleq s_i$ the *ith state on* $P$, and $P[i..]$ the *subpath* $s_i, \ldots, s_{n+1}$. Any state $s$ is defined to be *reachable* (in $G$) if there exists an $(s_I, s)$-path. Any path $P$ is a *cycle* whenever $P[0] = P[|P| - 1]$ and $1 < |P|$. Finally, any cycle $P$ is *accepting* if $P[i] \in \mathcal{A}$ for some $0 \leq i < |P|$.

#### 2.1 Nested Depth-First Search

Figure 1 presents a standard, sequential implementation of NDFS, consisting of two nested DFS searches: `dfsblue` and `dfsred`. The *blue search* processes successors recursively in DFS order, marking them blue when done on line 8. The colour cyan indicates a partially explored state, i.e., not all of its successors have been visited yet by the blue search. Just before backtracking from an accepting

```
1  void dfsblue(s)
2    s.color1 := cyan;
3    for t ∈ succ(s) do
4      if t.color1 = white then
5        dfsblue(t);
6    if s ∈ A then
7      dfsred(s);
8    s.color1 := blue;

9  void dfsred(s)
10   s.color2 := pink;
11   for t ∈ succ(s) do
12     if t.color1 = cyan then
13       report cycle; exit;
14     if t.color2 = white then
15       dfsred(t);
16   s.color2 := red;
```

Fig. 1: A standard sequential implementation of nested DFS.

state, `dfsblue` calls the *red search* on line 7, to report any accepting cycle. This colours a state red after processing its successors recursively on line 16. The pink colour denotes states that are only partially explored by `dfsred`[5].

It is straightforward to see that NDFS is *sound*, meaning that it only reports true accepting cycles. To see that NDFS is also *complete*, i.e., finds an accepting cycle if one exists, observe that `dfsred` will indeed be started from every accepting state. This in itself is not enough: the red search ignores states marked red in a previous call. It is essential that `dfsred` explores accepting states in the right order. The crucial insight is that `dfsred` only visits cyan and blue states and that accepting states coloured blue cannot be part of any accepting cycle.

The correctness of NDFS has been verified with Dafny [37]. We ported this correctness proof to VerCors as the basis for the verification of parallel NDFS.

#### 2.2 Parallel Nested Depth-First Search

A naive strategy for parallelising NDFS is *swarming* [18]: running several instances of NDFS in parallel, each working on a private set of colours. Swarmed NDFS tends to find accepting cycles faster, since its workers are expected to explore different parts of the input graph. The correctness of swarmed NDFS with respect to sequential NDFS is almost immediate, except for termination handling: workers only share information about the exit condition. We also verified swarmed NDFS in VerCors, as a stepping stone for verifying parallel NDFS.

Laarman et al. improve on the swarming algorithm by sharing information of the red search in the backtrack phase. Figure 2 presents the improved algorithm. Here every line of code is supposed to be executed atomically. The entry point is $\texttt{pndfs}(s_I, n)$, which spawns $n$ parallel instances of $\texttt{dfsblue}(s_I, tid)$ in the fashion of swarming. However, the red colourings are shared now, by which workers can guarantee that certain states are, or will be, sufficiently explored. So the red states can now be skipped in both the red search (line 19) *and* the blue search (line 4). PNDFS thus improves performance, since workers prune each other's search space. At the same time this significantly complicates the correctness argument, since workers may now prevent each other from finding accepting

[5] In the sequential algorithm, pink and red do not need to be distinguished, but having the distinction here makes the parallel version easier to explain.

```
1  void dfsblue(s, tid)
2    s.color[tid] := cyan;
3    for t ∈ succ(s) do
4      if t.color[tid] = white ∧ ¬t.red then
5        dfsblue(t, tid);
6    if s.acc ∧ ¬s.red then
7      s.count := s.count + 1;
8      dfsred(s, tid);
9    s.color[tid] := blue;
10 void pndfs(s, nthreads)
11   par tid = 0 to nthreads do
12     dfsblue(s, tid);
13   report no cycle;
14 void dfsred(s, tid)
15   s.pink[tid] := true;
16   for t ∈ succ(s) do
17     if t.color[tid] = cyan then
18       report cycle; exit all;
19     if ¬t.pink[tid] ∧ ¬t.red then
20       dfsred(t, tid);
21   if s.acc then
22     s.count := s.count − 1;
23     await s.count = 0;
24   s.pink[tid] := false, s.red := true;
```

Fig. 2: An implementation of parallel NDFS, where the red colours are shared.

cycles. Moreover, if multiple workers initiated `dfsred` from the same accepting state $s$, they must now finish their red search simultaneously for the algorithm to be correct. The **await** synchroniser on line 23 ensures this, by blocking thread execution until $s.count$—the number of workers in `dfsred`$(s, \cdot)$—reaches 0.

The original correctness argument of Laarman et al. relies on a complicated inductive invariant stating that not all accepting cycles can be missed due to pruning. However, this invariant is unsuitable for use in a (semi-)automated verifier. Section 3 discusses the verification of `pndfs` and provides a new invariant on the red colours that allows its correctness to be proven mechanically. It also discusses how our verification handles concurrency and thread synchronisation.

#### 2.3 Concurrency Verification with VerCors

Before discussing the actual verification, let us first briefly introduce VerCors, an automated program verifier for parallel programs. VerCors uses concurrent separation logic with permissions as its logical foundation. Its annotation language contains *fractional permission predicates* of the form $\mathsf{Perm}(s, \pi)$, in the style of Boyland [7], that capture the notion of ownership enforced by separation logic, where $s$ is a shared memory location (e.g., a class field) and $\pi \in (0, 1]_{\mathbb{Q}}$ a *fractional value*. The fractional permissions denote access rights: if $\pi = 1$ it denotes *write access* to $s$, whereas $\pi < 1$ denotes a *read access* to $s$. Sometimes $\mathsf{Perm}(s)$ is written as shorthand for $\exists\pi : \mathsf{Perm}(s, \pi)$, to indicate *some* ownership of $s$. Soundness of the underlying logic ensures that the total sum of permissions for any shared memory location does not exceed 1, which implies data race freedom.

In addition to ownership predicates, the annotation language supports the $\ast\!\!\ast$ connective, which is the *separating conjunction* of separation logic. The assertion $P \ast\!\!\ast Q$ expresses that the ownerships captured by $P$ and $Q$ are *disjoint*, e.g., it is disallowed that both express write access to the same shared location. Ownership predicates can be *split* into disjoint parts and be *combined* as follows:

$$\mathsf{Perm}(s, \pi_1 + \pi_2) \iff \mathsf{Perm}(s, \pi_1) \ast\!\!\ast\ \mathsf{Perm}(s, \pi_2)$$

A standard pattern in concurrency verification is to split and distribute the ownership of all shared memory over *threads* and *locks*. Clarifying the latter; in case multiple threads need to write to a common footprint of shared memory, the ownerships to this footprint are typically protected by a *resource invariant*. Threads can then only use the resources protected by this invariant when they execute *atomic* instructions (i.e., when no other threads can interfere). For more details we refer to the standard papers on concurrent separation logic [34,8,44].

## 3 Automated Verification of Parallel NDFS

This section elaborates on the verification of `pndfs` with VerCors [35]. Section 3.1 presents and discusses our new correctness argument for `pndfs`, which includes the new invariant on the red colours and a proof of its correctness. Sections 3.2 and 3.3 discuss the mechanisation of this proof in VerCors.

### 3.1 Correctness of `pndfs`

The soundness proof of `pndfs` is not very different from the soundness argument of sequential NDFS: every time **report cycle** is executed, a witness cycle can be found. The main challenge lies in proving completeness, i.e., proving that if there exists any accepting cycle, `pndfs` will report it. This is difficult since workers can *obstruct* each other's red searches and thereby prevent the detection of accepting cycles. This section proposes a new key invariant and completeness proof that is suitable for deductive verification.

We start by introducing a number of low-level invariants on the local configurations of colours that can arise during a run of `pndfs`. Let $\mathit{Cyan}_{tid}$ be the set of cyan-coloured states $\{s \in \mathcal{S} \mid s.\mathit{color}[\mathit{tid}] = \mathsf{cyan}\}$ private to worker $\mathit{tid}$, and likewise for $\mathit{White}_{tid}$, $\mathit{Blue}_{tid}$ and $\mathit{Pink}_{tid}$. Moreover, let $\mathit{Red}$ be the set of globally red states, and $\mathsf{succ}(X) \triangleq \bigcup_{s \in X} \mathsf{succ}(s)$ the *successor set* of a given set $X \subseteq \mathcal{S}$.

**Lemma 1.** `pndfs` *maintains the following global invariants during execution:*

*1.1.* $\forall \mathit{tid} : \mathsf{succ}(\mathit{Blue}_{tid} \cup \mathit{Pink}_{tid}) \subseteq \mathit{Blue}_{tid} \cup \mathit{Cyan}_{tid} \cup \mathit{Red}$
*1.2.* $\mathsf{succ}(\mathit{Red}) \subseteq \mathit{Red} \cup \bigcup_{tid}(\mathit{Pink}_{tid} \setminus \mathit{Cyan}_{tid})$
*1.3.* $\forall \mathit{tid} : \mathcal{A} \cap \mathit{Blue}_{tid} \subseteq \mathit{Red}$
*1.4.* $\forall \mathit{tid} : \mathcal{A} \cap \mathit{Pink}_{tid} \subseteq \mathit{Cyan}_{tid}$
*1.5.* $\forall \mathit{tid} : \mathit{Pink}_{tid} \subseteq \mathit{Blue}_{tid} \cup \mathit{Cyan}_{tid}$
*1.6.* $\forall \mathit{tid} : |\mathcal{A} \cap \mathit{Pink}_{tid}| \leq 1$

*Proof.* The proof basically checks their preservation by each line of the program.

Invariants *1.1–1.5* are reused from [25], whereas *1.6* is new and needed for the new completeness proof. Proving completeness amounts to proving that not all reachable accepting cycles can be missed due to search space pruning. To help proving this, we identify a new class of paths, which we call *tid-special paths*.

**Definition 1 (Special path).** *Any path* $P = s_0, \ldots, s_{n+1}$ *is defined to be tid*-special *if* $s_0 \in Pink_{tid}$, $s_{n+1} \in Cyan_{tid}$, *and none of the states on* $P$ *are red, i.e.,* $s_k \notin Red$ *for every* $k$ *such that* $0 \leq k \leq n+1$.

Any path $P$ is *special* if $P$ is *tid*-special for some worker *tid*. Intuitively, the existence of a *tid*-special path during execution of `pndfs` means that (*i*) worker *tid* is doing a red search, since it has pink states, and (*ii*) this worker will eventually find an accepting cycle, unless other workers obstruct this path. Thus the above definition allows to formally define obstruction: a worker *tid* is *obstructed* (will miss an accepting cycle) if any state on a *tid*-special path is coloured red.

Our main strategy for proving completeness involves showing that every time a worker gets obstructed, a new special path can be found. A direct consequence of this is that not all accepting cycles can be missed: upon termination of `pndfs`, there are no more cyan or pink states. To help prove this, we use the following property (taken from [25], but rephrased to handle our special paths), that allows to find special paths by using the colouring invariants.

**Lemma 2.** *If invariants 1.1–1.6 are satisfied, then every path* $P = s_0, \ldots, s_{n+1}$ *with* $s_0 \in Red$ *and* $s_{n+1} \in \mathcal{A} \setminus Red$ *contains a special subpath.*

*Proof.* The original handwritten proof from [25] shows that this lemma follows from invariants *1.1–1.6*, by induction on $P$. □

The original completeness proof of [25] performs induction on the number of obstructed accepting cycles, to show the absence of such cycles upon termination as a result of Lemma 2. However, such an argument is out of reach for Hoare-style reasoning, since it is not an *inductive* invariant. We propose a new invariant that *is* inductive, which builds on the insight that, under certain colouring conditions, new special paths can always be found when workers get obstructed, as is shown by Lemma 3. In particular, `pndfs` guarantees that if there exists a special path before executing line 24, then there also exists a special path after its execution.

**Lemma 3.** *For any non-red state* $r \in \mathcal{S} \setminus Red$ *that is on a tid-special path, if:*

*i.* $r \in \mathcal{A} \implies \mathsf{succ}(r) \subseteq Red$, *and*
*ii.* $r \in \mathcal{A} \cap Pink_{tid} \implies Pink_{tid} = \{r\}$,

*then there still exists a special path after adding* $r$ *to Red.*

*Proof.* Let $P = s_0 \ldots s_{n+1}$ be a *tid*-special path and assume that $r$ is on $P$, so that $r = s_\ell$ for some $\ell$ such that $0 \leq \ell \leq n+1$. Since $Pink_{tid} \neq \emptyset$, worker *tid* is performing `dfsred` that was started from some accepting state $a \in \mathcal{A} \cap Pink_{tid}$. Then $a \neq r$, as otherwise $s_0 = a$ due to *ii.*, which by *i.* would contradict that $P$ is special. Moreover, since $s_{n+1} \in Cyan_{tid}$ there exists a $(s_{n+1}, a)$-path $Q$ (this is a standard property of `dfsblue`; the path $Q$ must be on the recursive call stack). Then Lemma 2 applies on the path $s_\ell, \ldots, s_{n+1}, Q[1..]$ and gives a new special path when considering $Red \cup \{r\}$ as the new set of red states. □

Lemma 3 implies that every time an accepting cycle is missed due to pruning, there is always another accepting cycle that will eventually be reported. This is enough to establish completeness of `pndfs`, via the following key invariant.

**Lemma 4.** *The* `pndfs` *algorithm maintains the global invariant that either:*

*4.1. All reachable accepting cycles contain an accepting state that is not red; or*
*4.2. There exists a special path.*

*Proof.* The interesting case is showing that this invariant remains preserved after making a non-red state $s \in \mathit{Pink}_{tid} \setminus \mathit{Red}$ red (on line 24 of Fig. 2), by some worker *tid* that is doing a red search from some accepting state $a \in \mathcal{A} \cap \mathit{Pink}_{tid}$.

– Suppose $s \notin \mathcal{A}$. If $s$ is on a special path, then Invariant *4.2* is reestablished due to Lemma 3, and otherwise the key invariant remains preserved.
– Suppose $s \in \mathcal{A}$. Then $s = a$ by Invariant *1.6*. Since worker *tid* is about to finish its red exploration, we have that (†) $\mathit{Pink}_{tid} = \{s\}$ (i.e., all other pink states have been fully explored) and consequently that (‡) $\mathsf{succ}(s) \subseteq \mathit{Red}$. Furthermore, due to the **await** $s$ instruction on line 23 we have that (†) and (‡) hold for *all* workers that are doing a red exploration that involves $s$. If $s$ is on a special path, then Invariant *4.2* is reestablished due to Lemma 3. So now suppose that $s$ is on an accepting cycle $P$. Without loss of generality, assume that $P[0] = s$. Then (‡) implies that $1 < |P|$ and that $P[1] \in \mathit{Red}$. Thus Lemma 3 applies on the path $P[1..]$ to establish Invariant *4.2*. □

The next theorem shows how Lemma 4 allows deriving completeness of parallel NDFS. In particular, it shows that no accepting cycles can exist when all threads have terminated, in which case all the theorem's premises are fulfilled.

**Theorem 1.** *If for every worker tid it holds that* $\mathit{Pink}_{tid} = \emptyset$, $\mathit{Cyan}_{tid} = \emptyset$ *and* $s_I \in \mathit{Blue}_{tid}$, *then there does not exist a reachable accepting cycle.*

*Proof.* Towards a contradiction, suppose that there exists an accepting cycle $P$ that is reachable via an $(s_I, P[0])$-path $Q$. Due to the theorem's premises no special paths can exist, and therefore by Lemma 4 there is an accepting state on $P$ that is not red. Without loss of generality, assume that (†) $P[0] \in \mathcal{A} \setminus \mathit{Red}$. Since $Q[0] \in \mathit{Blue}_0$ (since there is at least one worker), by induction on $Q$ together with Lemma 1 we have that $P[0] \in \mathit{Red}$, which contradicts (†). □

All the above invariants and proof steps have been encoded in VerCors, which was highly non-trivial. While mechanising the proofs, many implicit proof steps had to be made explicit. Section 3.3 further details the proof mechanisation.

#### 3.2 Encoding of `pndfs` in VerCors

Graph structures are notoriously difficult to handle in separation logics, as they usually rely on pointer aliasing, which complicates ownership handling and prevents easy use of the frame rule [38]. However, since automata have a fixed and finite set of states, we can overcome this limitation by representing the input automata as an $|\mathcal{S}| \times |\mathcal{S}|$ *adjacency matrix*. This does not impose serious restrictions: other automata encodings can be transformed at the specification level to an adjacency matrix, e.g., via model fields in the style of JML [11,29]. The suitability of adjacency matrices for deductive verification is confirmed by [24].

```
1  enum Color {white, cyan, blue};
2  int N; // the number of automata states (equal to |S|)
3  int nthreads; // the total number of participating workers
4  bool[N][N] G; // adjacency matrix representation of the input automaton
5  bool[N] acc; // the encoding of the set of accepting states
6  Color[nthreads][N] color; // the colour sets for dfsblue (one for each thread)
7  bool[nthreads][N] pink; // the pink colour sets for dfsred (one per thread)
8  bool[N] red; // the global set of red colourings
9  bool abort; // global termination flag
10
11 resource resource_invariant ≜ ···; // full definition is deferred to Fig. 4.
12
13 bool Path(int s, int t, seq⟨int⟩ P) ≜ // the encoding of (s,t)-paths in G
14   0 ≤ s,t < N ∧ 0 < |P| ∧ P[0] = s ∧ P[|P| − 1] = t ∧
15     (∀i : 0 ≤ i < |P| ⇒ 0 ≤ P[i] < N) ∧ (∀i : 0 ≤ i < |P|−1 ⇒ G[P[i]][P[i+1]]);
16 bool Path(seq⟨int⟩ P) ≜ 0 < |P| ∧ Path(P[0], P[|P| − 1], P);
17 bool ExPath(int s, int t, int n) ≜ ∃P : n ≤ |P| ∧ Path(s, t, P);
18 bool SpecialPath(seq⟨int⟩ P, int tid) ≜ // the encoding of tid-special paths
19   pink[tid][P[0]] ∧ color[tid][P[|P| − 1]] = cyan ∧ ∀i : 0 ≤ i < |P| ⇒ ¬red[P[i]];
20 bool ExSpecialPath(int tid) ≜ ∃P : 1 < |P| ∧ Path(P) ∧ SpecialPath(P, tid);
21
22 /* An excerpt of the top-level contract (further discussed in Section 3.3). */
23 ensures \result ⇒ (∃a : 0 ≤ a < N ∧ acc[a] ∧ ExPath(s_I, a, 1) ∧ ExPath(a, a, 2));
24 ensures (∃a : 0 ≤ a < N ∧ acc[a] ∧ ExPath(s_I, a, 1) ∧ ExPath(a, a, 2)) ⇒ \result;
25 bool pndfs(int s_I);
```

Fig. 3: The automata representation and an excerpt of `pndfs`'s top-level contract.

Figure 3 shows the encoding of the input automaton $G$ in VerCors. The thread-local colour sets are represented as matrices of dimension $\mathit{nthreads} \times |\mathcal{S}|$, so that each thread $\mathit{tid}$ uses $\mathit{color}[\mathit{tid}][\cdot]$ and $\mathit{pink}[\mathit{tid}][\cdot]$ to administrate their (local) status of exploration. The sets of red and accepting states are shared between threads and thus encoded as $|\mathcal{S}|$-sized Boolean arrays. The $\mathsf{succ}$ function can now be defined such that $t \in \mathsf{succ}(s)$ whenever $G[s][t]$ is true for every $0 \leq s, t < N$.

This encoding of automata, together with an encoding of the definition of paths (on lines 13–17) is sufficient to express the main correctness property that is proven by VerCors. More specifically, line 23 expresses *soundness*: a positive return value indicates the existence of an accepting cycle. Line 24 expresses *completeness*: if there exists an accepting cycle, then `pndfs` returns positively.

**Atomic operations.** The handwritten correctness argument of [25] for Figure 2 assumes that all program lines are executed atomically. This is reflected in the VerCors encoding: all updates to shared memory are made within atomic operations, which specification-wise all give access to the same shared resources. For example, the assignment $s.\mathit{pink}[\mathit{tid}] := \mathit{true}$ on line 15 (Fig. 2) is implemented as the atomic operation "**atomic** { $\mathit{pink}[\mathit{tid}][s] := \mathit{true}$ }". On the specification level, the atomic sub-program receives all the missing access rights required for the assignment, which are otherwise protected by the resource invariant declared on line 11 (Fig. 3). The exact definition of `resource_invariant` is deferred to §3.3, and the type **resource** is the type of separation logic assertions. Moreover, the **await** instruction on line 23 (Fig. 2) is implemented as a busy while-loop that only stops when $s.count = 0$, which is checked atomically in every iteration.

**Termination handling.** The pseudocode in Figure 2 uses an "**exit all**" command to terminate all threads when an accepting cycle has been found. However, this mechanism was left implicit. Our formalisation in VerCors makes the termination system explicit: it consists primarily of a global *abort* flag that is declared on line 9 in Figure 3. All workers regularly poll this flag to determine whether they continue or not. The *abort* flag is set to true by the main thread—the thread that started `pndfs` and spawned all worker threads on line 11 of Fig. 2—as soon as one of the workers returns with an accepting cycle.

#### 3.3 Verification of `pndfs` in VerCors

One major challenge of concurrency verification is finding a proper distribution of shared-memory ownership, that allows proving memory safety as well as any functional properties of interest. This section starts by discussing how we distribute the ownership of the input automaton over threads and the resource invariant, in such a way that Invariants *1.1–1.6* and *4.1–4.2* can be encoded.

To prove the preservation of these invariants after every computation step, auxiliary bookkeeping is needed on the specification level. For example, to mechanise the proof of Lemmas 3 and 4 we need to make explicit that all workers *tid* with $Pink_{tid} \neq \emptyset$ are doing a red search that was started from some root state $a \in \mathcal{A} \cap Pink_{tid}$. This auxiliary bookkeeping is maintained in the resource invariant, via *auxiliary ghost state*, which is explained later. Finally, we give the fully annotated version of `pndfs` and explain how completeness is proven from Lemma 4, by applying the VerCors encoding of Theorem 1.

**Ownership distribution.** We start by explaining how the ownership of the automaton encoding (lines 2–8 in Fig. 3) is distributed among workers and the resource invariant. First observe that all colouring invariants express *global properties* that span over $(i)$ the shared *red* colourings, as well as $(ii)$ the local configurations $color[tid]$ and $pink[tid]$ of every worker *tid*. To define the ownership distribution for $(i)$, observe that the only way to distribute the access rights to *red* to enable all threads to regain write access, is to let the resource invariant protect full ownership of *red*. The resource invariant therefore fully captures the properties about red states expressed in Lemmas 1 and 4. However, to be able to specify that, it also requires partial ownership of all thread-local colourings.

Figure 4 presents the full resource invariant, that includes: access rights to both global and thread-local colourings on lines 2–4; the encoding of Lemma 1 on lines 10–17 and 22; and the encoding of Lemma 4 on lines 30–32. In addition, the resource invariant holds partial ownership of the *abort* flag on line 8, to ensure that global termination is only announced when an accepting cycle is found.

```
resource resource_invariant ≜
   Perm(N) ∗∗ Perm(nthreads) ∗∗ Perm(G) ∗∗ Perm(acc) ∗∗
   (∀tid, s : Perm(color[tid][s], 1/2) ∗∗ Perm(pink[tid][s], 1/2)) ∗∗
   (∀s : Perm(red[s], 1)) ∗∗
   termination() ∗∗ colourings() ∗∗ dfsred_status() ∗∗ keyinvariant(); resource termination() ≜ // Resources for termination handling.
   Perm(abort, 1/2) ∗∗ abort ⇒ ∃s : acc[s] ∧ ExPath(s_I, s, 1) ∧ ExPath(s, s, 2); resource colourings() ≜ // The low-level colouring invariant encodings.
   ∀tid, s : (color[tid][s] = blue ∨ pink[tid][s]) ⇒ ∀s′ ∈ succ(s) :
      color[tid][s′] = blue ∨ color[tid][s′] = cyan ∨ red[s′] ∗∗ // Inv. 1.1
   ∀s : red[s] ⇒ ∀s′ ∈ succ(s) :
      red[s′] ∨ ∃tid : pink[tid][s′] ∧ color[tid][s′] ≠ cyan ∗∗ // Inv. 1.2
   ∀tid, s : (acc[s] ∧ color[tid][s] = blue) ⇒ red[s] ∗∗ // Inv. 1.3
   ∀tid, s : (acc[s] ∧ pink[tid][s]) ⇒ color[tid][s] = cyan ∗∗ // Inv. 1.4
   ∀tid, s : pink[tid][s] ⇒ (color[tid][s] = cyan ∨ color[tid][s] = blue); // 1.5

/∗ Auxiliary ghost state for proving Lemma 3 and preserving Inv. 4. ∗/
resource dfsred_status() ≜ ∀tid : (
   Perm(exploringred[tid], 1/2) ∗∗ Perm(redroot[tid], 1/2) ∗∗ Perm(waiting[tid], 1/2) ∗∗
   ∀s : pink[tid][s] ⇒ (exploringred[tid] ∧ (acc[s] ⇒ s = redroot[tid])) ∗∗ // 1.6
   exploringred[tid] ⇒ acc[redroot[tid]] ∧
      (∀s : pink[tid][s] ⇒ ExPath(redroot[tid], s, 1)) ∧
      (∀s : color[tid][s] = cyan ⇒ ExPath(s, redroot[tid], 1)) ∧
      (¬waiting[tid] ⇒ ¬red[redroot[tid]]) ∧
      (waiting[tid] ⇒ ∀s : pink[tid][s] ⇔ s = redroot[tid]))

/∗ The encoding of Lemma 4, from which completeness of pndfs follows. ∗/
resource keyinvariant() ≜
   (∀s : acc[s] ∧ ExPath(s_I, s, 1) ∧ ExPath(s, s, 2) ⇒ ¬red[s]) ∨
   (∃tid : ExSpecialPath(tid));
```

Fig. 4: The full definition of the resource invariant. Several bound checks have been omitted for presentational clarity.

Observe that the resource invariant holds a lot of quantified information. As a result, we experienced that proving the reestablishment of `resource_invariant` after finishing **atomic**s is expensive performance-wise. To make verification more efficient, we extracted all atomic operations (e.g., colour updates) into separate methods and prove their contracts in a function-modular way. This improves performance, as it cuts the problem of verifying `dfsred` and `dfsblue` into smaller sub-problems that are individually more manageable for the SMT solver.

Finally, Figure 5 presents an excerpt of the contract of `dfsblue`, which shows the ownership pattern of all threads. Notably, every thread *tid* receives the remaining ownership of *color*[*tid*] and *pink*[*tid*] on line 4. Thus threads can always read from their thread-local colour fields, and may write to them while doing so

1 **context** $\mathsf{Perm}(N) \ast\!\ast\, \mathsf{Perm}(\mathit{nthreads}) \ast\!\ast\, \mathsf{Perm}(G) \ast\!\ast\, \mathsf{Perm}(\mathit{acc})$;
2 **context** $0 \leq s < N$;
3 **context** $0 \leq \mathit{tid} < \mathit{nthreads}$;
4 **context** $\forall t : 0 \leq t < N \Rightarrow \mathsf{Perm}(\mathit{color}[\mathit{tid}][t], \frac{1}{2}) \ast\!\ast\, \mathsf{Perm}(\mathit{pink}[\mathit{tid}][t], \frac{1}{2})$;
5 **requires** $\mathit{color}[\mathit{tid}][s] = \mathsf{white}$;
6 **requires** $\forall t : (0 \leq t < N \wedge \mathit{color}[\mathit{tid}][t] = \mathsf{cyan}) \Rightarrow \mathsf{ExPath}(t, s, 1)$;
7 **ensures** $\backslash\mathbf{result} \Rightarrow \exists a : 0 \leq a < N \wedge \mathit{acc}[a] \wedge \mathsf{ExPath}(s_I, a, 1) \wedge \mathsf{ExPath}(a, a, 2)$;
8 **ensures** $\neg\backslash\mathbf{result} \Rightarrow \forall t : \mathit{color}[\mathit{tid}][t] = \mathsf{cyan} \Leftrightarrow \backslash\mathbf{old}(\mathit{color}[\mathit{tid}][t]) = \mathsf{cyan}$;
9 **ensures** $\neg\backslash\mathbf{result} \Rightarrow \mathit{pink}[\mathit{tid}] = \backslash\mathbf{old}(\mathit{pink}[\mathit{tid}]) \wedge \mathit{color}[\mathit{tid}][s] = \mathsf{blue}$;
10 **bool** `dfsblue(`$s$, $\mathit{tid}$`)`
11 $\quad \cdots$

Fig. 5: The ownership specification in the contract `dfsblue` for thread *tid*. Annotations of the form **context** $P$ abbreviate **requires** $P$; **ensures** $P$.

atomically. This distribution of ownership matches with the encoding of atomic operations discussed earlier. Line 7 expresses soundness of `dfsblue`, captured in the resource invariant (line 8 of Fig. 4) on global termination. This allows to deduce soundness of `pndfs` from the resource invariant, after all threads have terminated as result of the detection of an accepting cycle.

**Auxiliary ghost state.** As mentioned earlier, to prove that `pndfs` also *preserves* the (encodings of) Invariants *1.1–1.6* and *4.1–4.2* after every computation step, additional ghost state needs to be maintained. In particular, we need to make explicit that every worker *tid* with $\mathit{Pink}_{\mathit{tid}} \neq \emptyset$ is doing a `dfsred` search that was started from some root state $a \in \mathcal{A} \cap \mathit{Pink}_{\mathit{tid}}$. In addition, the proof of Lemma 3 needs that there exists an $(s, a)$-path for every $s \in \mathit{Cyan}_{\mathit{tid}}$. To prove the preservation of Lemma 4 we also need that, if worker *tid* is not yet executing the **await** instruction, we have that $a \notin \mathit{Red}$, and otherwise that $\mathit{Pink}_{\mathit{tid}} = \{a\}$.

This extra information is encoded in the loop invariant on lines 20–27 (Figure 4), via three *ghost arrays*, named *exploringred*, *redroot* and *waiting*. Firstly, *exploringred* administrates which workers are doing a red search. For verification purposes we added ghost code to the program, to set $\mathit{exploringred}[\mathit{tid}]$ to true whenever `dfsred(`$a$, $\mathit{tid}$`)` is invoked by worker *tid* from a blue search, and back to false whenever `dfsred(`$a$, $\mathit{tid}$`)` returns. Secondly, *redroot* stores the root state on which `dfsred` was invoked. Finally, *waiting* administrates which workers are executing an **await** instruction. These three ghost arrays together are closely related to the $s.\mathit{count}$ fields in the program of Figure 2, via the following invariant: $\forall s : s.\mathit{count} = |\{\mathit{tid} \mid \mathit{exploringred}[\mathit{tid}] \wedge \mathit{redroot}[\mathit{tid}] = s \wedge \neg\mathit{waiting}[\mathit{tid}]\}|$.

Establishing that `pndfs` adheres to the invariants in Lemmas 1 and 4 was highly non-trivial and required various complex auxiliary lemmas to be encoded and proven. These are all encoded in VerCors as *ghost methods*: side-effect-free helper methods on which the lemma is encoded in the method's contract [21,22]. Induction proofs, for example, are encoded using either loop invariants or recursion. Application of a lemma then translates to a function call on the specification level. The proofs in Section 3.1 are all encoded and applied in this way.

```
1  context Perm(N) ∗∗ Perm(nthreads) ∗∗ Perm(G) ∗∗ Perm(acc) ∗∗ Perm(abort, 1/2);
2  context ∀tid, s : Perm(color[tid][s], 1/2) ∗∗ Perm(pink[tid][s], 1/2);
3  context ∀tid : Perm(exploringred[tid], 1/2) ∗∗ Perm(redroot[tid], 1/2);
4  context ∀tid : Perm(waiting[tid], 1/2);
5  context 0 ≤ s_I < N;
6  requires ∀tid, s : ¬exploringred[tid] ∧ color[tid][s] = white ∧ ¬pink[tid][s];
7  ensures \result ⇒ (∃a : acc[a] ∧ ExPath(s_I, a, 1) ∧ ExPath(a, a, 2));
8  ensures (∃a : acc[a] ∧ ExPath(s_I, a, 1) ∧ ExPath(a, a, 2)) ⇒ \result;
9  bool pndfs(s_I)
10   par tid = 0 to nthreads
11     context Perm(N) ∗∗ Perm(nthreads) ∗∗ Perm(G) ∗∗ Perm(acc);
12     context ∀s : Perm(color[tid][s], 1/2) ∗∗ Perm(pink[tid][s], 1/2);
13     context Perm(term[tid], 1/2) ∗∗ Perm(exploringred[tid], 1/2);
14     context Perm(redroot[tid], 1/2) ∗∗ Perm(waiting[tid], 1/2);
15     requires ¬exploringred[tid] ∧ ∀s : color[tid][s] = white ∧ ¬pink[tid][s];
16     ensures ¬abort ⇒ ∀s : color[tid][s] ≠ cyan ∧ ¬pink[tid][s];
17     ensures ¬abort ⇒ color[tid][s_I] = blue;
18    do
19     bool found := dfsblue(s_I, tid);
20     if found then
21       atomic { abort := true; } // initiate global termination.
22   atomic { if ¬abort then theorem_one() }; // apply Thm. 1's encoding.
23   return abort;
```

Fig. 6: The annotated version of `pndfs`, extending the excerpt given in Figure 3.

**Correctness of `pndfs`.** Figure 6 gives the annotated version of `pndfs`[6] that extends the excerpt given earlier, in lines 23–25 of Figure 3. The main thread requires partial ownership of all thread-local colour fields on line 2 and distributes these over the appropriate threads on line 12. The contract associated to the parallel block (lines 11–17) is called an *iteration contract* and assigns pre- and postconditions to every parallel instance. For more details on iteration contracts we refer to [5]. Most importantly, the iteration contract of each thread holds enough resources to satisfy all the preconditions of `dfsblue`, on line 19.

Soundness of `pndfs` (line 7) is proven as follows. Suppose that all threads have terminated and *abort* has been set to *true*. In that case, the resource invariant states that an accepting cycle has been found. This information can be retrieved by briefly obtaining the resource invariant in *ghost code* on line 22, which directly allows to deduce soundness. Note that this information is not lost upon releasing the resource invariant, as it is a Boolean property and thus duplicable.

To prove completeness, suppose that *abort* is still false when all workers have terminated. This implies that $Pink_{tid} = \emptyset$ and $Cyan_{tid} = \emptyset$ for every worker *tid* (line 16), as well as $s_I \in Blue_{tid}$ (line 17), since all threads started their blue

[6] Observe that every thread reads *abort* in their contract on lines 16–17, even though they do not have the required access rights to do so. This is resolved by adding some auxiliary ghost state, but this is omitted for presentational clarity.

```
1  void dfsblue(s, tid)
2    s.color[tid] := cyan;
3    for t ∈ succ(s) do
4      if t.color[tid] = cyan then
5        if s.acc ∨ t.acc then
6          report cycle; exit all;
7      if t.color[tid] = white then
8        if ¬t.red then
9          dfsblue(t, tid);
10   if s.acc ∧ ¬s.red then
11     s.count := s.count + 1;
12     dfsred(s, tid);
13   s.color[tid] := blue;
```

(a) The "early cycle detection" extension

```
1  void dfsblue(s, tid)
2    allred := true;
3    s.color[tid] := cyan;
4    for t ∈ succ(s) do
5      if t.color[tid] = white then
6        if ¬t.red then
           dfsblue(t, tid);
7      if ¬t.red then allred := false;
8    if allred then
9      await s.count = 0;
10     s.red := true;
11   else if s.acc ∧ ¬s.red then
12     s.count := s.count + 1;
13     dfsred(s, tid);
14   s.color[tid] := blue;
```

(b) The "all-red" extension

Fig. 7: Two extensions (highlighted grey) to `dfsblue` that improve work sharing.

search from $s_I$. Combining this information with the information in the resource invariant allows one to prove all the premises of Theorem 1. Therefore its ghost method encoding can be applied on line 22, from which completeness is derived.

The encoding of parallel NDFS in VerCors [35] comprises roughly 2500 lines of code (of which ∼85% is proof overhead), which includes the mechanisation of all proof steps described in §3.1. The verification time is about 140s, measured on a Macbook with an Intel Core i5 CPU with 2,9 GHz, and 8Gb memory.

### 4 Optimisations

One major benefit of mechanically verified code is that optimisations can be applied with full confidence. Without verification, changes to critical code are often avoided, to ensure that no errors are introduced. A verified algorithm allows to apply optimisations easily, as these often do not change the outer contract, at most requiring only minor adaptions to the invariants. We illustrate this with two optimisations, for which [25] experimentally demonstrated improved speedup.

"Early cycle detection" checks already in the blue search if an accepting cycle is closed, cf. lines 4–6 in Figure 7a. It is known that for weak LTL properties, all accepting cycles will be found in the blue search when applying early cycle detection. To show that this optimisation indeed preserves all invariants, we simply inserted these 3 lines in the VerCors specification. The proof introduces a case distinction on whether $s$ or $t$ is accepting and constructs a witness path. This adds another 10 lines: two for the case distinction and four in each branch to show that a witness accepting cycle exists. Collectively, these extra 13 lines constitute indeed very little effort to prove this particular optimisation correct.

The second optimisation, called "all-red", checks if all successors of $s$ became red during the blue search (lines 2 and 7 in Figure 7b). If so, we can mark $s.red$

early (lines 8–10). This optimisation is important, since it allows the global red colour to spread even in portions of the graph that are not under an accepting state, thereby allowing more pruning. However, this optimisation only preserves the invariants if we wait until $s.count = 0$ (on line 9). This test was erroneously omitted in [25][7]. Fortunately, the version in Figure 7b is correct, which has now been checked in VerCors in a straightforward manner.

### 5 Conclusion

This paper presents the first automated deductive verification of a parallel graph algorithm: we verified soundness and completeness of parallel nested depth-first search using VerCors. We also show that this mechanisation is helpful in quickly discovering whether optimisations of the algorithm preserve its correctness.

Many of the presented verification techniques, e.g., the use of separate contracts for single statements, the way we handle termination, and the construction of explicit witnesses through ghost variables, will be useful for the verification of other similar algorithms. Moreover, our encoding of parallel nested DFS closely resembles the implementation of such an algorithm in mainstream programming languages like C++ and Java. It would be interesting to investigate how our VerCors encoding can be automatically deployed on multi-core architectures, for example to enable comparing its performance and scalability with LTSmin.

There are many possibilities to extend the line of research on the verification of parallel model checking algorithms initiated in this paper. First, one may consider to extend the scope of this verification closer towards the actual efficient C-implementation in LTSmin. This would involve verifying the underlying concurrent hash table to store visited states (a simplified version of which has been verified before with VerCors [1]), the encoding of the colours as "bits" in the hash table buckets, and the use of CAS to manipulate these bits.

One might consider alternative parallel NDFS versions, notably [15], which shares the blue colour, invoking a repair procedure when the depth-first order is violated. Both algorithms have been reconciled in [14], sharing both blue and red. This work could be extended to a wealth of other optimisations like partial-order reduction, or other parallel model checking algorithms, for example [26,4,40].

Our work can be considered as a first step towards a library for the verification of graph-based (multi-core) model checking algorithms. It will be an interesting line of future work to continue this: developing a full-fledged verification library for common subtasks, like graph manipulations and termination detection.

**Acknowledgments and data availability statement.** This work is partially supported by the NWO VICI 639.023.710 Mercedes project and by the NWO TOP 612.001.403 VerDi project. The datasets for this case study are available at: https://doi.org/10.4121/uuid:36c00955-5574-44d9-9b26-340f7a1ea03b.

[7] Wan Fokkink and his students Stefan Vijzelaar and Pieter Hijma already found in 2012 that the "all-red" extension required an extra check '**await** $s.count = 0$' in [25], and wondered whether '**await** $s.count \leq 1$' would be sufficient. Independently, Akos Hajdu reported this omission in 2015.

## References

[1] A. Amighi, S. Blom, and M. Huisman. Resource Protection Using Atomics - Patterns and Verification. In *APLAS*, pages 255–274, 2014. 10.1007/978-3-319-12736-1_14.

[2] J. Barnat, V. Bloemen, A. Duret-Lutz, A. Laarman, L. Petrucci, J. van de Pol,and E. Renault. Parallel Model Checking Algorithms for Linear-Time Temporal Logic. In *Handbook of Parallel Constraint Reasoning*, pages 457–507. Springer, 2018. 10.1007/978-3-319-63516-3_12.

[3] J. Barnat and I. Cerná. Distributed breadth-first search LTL model checking. *Formal Methods in System Design*, 29(2):117–134, 2006. 10.1007/s10703-006-0009-y.

[4] V. Bloemen, A. Laarman, and J. van de Pol. Multi-core On-the-fly SCC Decomposition. In *PPoPP*, pages 1–12. ACM, 2016. 10.1145/2851141.2851161.

[5] S. Blom, S. Darabi, and M. Huisman. Verification of Loop Parallelisations. In *FASE*, pages 202–217. Springer, 2015. 10.1007/978-3-662-46675-9_14.

[6] S. Blom, S. Darabi, M. Huisman, and W. Oortwijn. The VerCors Tool Set: Verification of Parallel and Concurrent Software. In *iFM*, LNCS, pages 102–110. Springer, 2017. 10.1007/978-3-319-66845-1_7.

[7] J. Boyland. Checking Interference with Fractional Permissions. In *SAS*, LNCS, pages 55–72. Springer, 2003. 10.1007/3-540-44898-5_4.

[8] S. Brookes. A Semantics for Concurrent Separation Logic. *Theoretical Computer Science*, 375(1–3):227–270, 2007. 10.1016/j.tcs.2006.12.034.

[9] J. Brunner and P. Lammich. Formal Verification of an Executable LTL Model Checker with Partial Order Reduction. *Journal of Automated Reasoning*, 60(1):3–21, 2018. 10.1007/s10817-017-9418-4.

[10] R. Chen, C. Cohen, J. Lévy, S. Merz, and L. Théry. Formal Proofs of Tarjan’s Algorithm in Why3, Coq, and Isabelle. *CoRR*, 2018. URL: http://arxiv.org/abs/1810.11979.

[11] Y. Cheon, G. Leavens, M. Sitaraman, and S. Edwards. Model Variables: Cleanly Supporting Abstraction in Design by Contract: Research Articles. *Software–Practice and Experience*, 35(6):583–599, 2005. 10.1002/spe.v35:6.

[12] E. Clarke, T. Henzinger, H. Veith, and R. Bloem, editors. *Handbook of Model Checking*. Springer, 2018. 10.1007/978-3-319-10575-8.

[13] C. Courcoubetis, M. Vardi, P. Wolper, and M. Yannakakis. Memory-Efficient Algorithms for the Verification of Temporal Properties. *Formal Methods in System Design*, 1(2–3):275–288, 1992. 10.1007/BF00121128.

[14] S. Evangelista, A. Laarman, L. Petrucci, and J. van de Pol. Improved Multi-Core Nested Depth-First Search. In *ATVA*, LNCS, pages 269–283. Springer, 2012. 10.1007/978-3-642-33386-6_22.

[15] S. Evangelista, L. Petrucci, and S. Youcef. Parallel Nested Depth-First Searches for LTL Model Checking. In *ATVA*, LNCS, pages 381–396. Springer, 2011. 10.1007/978-3-642-24372-1_27.

[16] A. Griggio, M. Roveri, and S. Tonetta. Certifying Proofs for LTL Model Checking. In *FMCAD*, pages 225–233, 2018. 10.23919/FMCAD.2018.8603022.

[17] G. Holzmann. The Model Checker SPIN. *IEEE Transactions on Software Engineering*, 23(5):279–295,1997. 10.1109/32.58852110.1109/32.588521.

[18] G. Holzmann, R. Joshi, and A. Groce. Swarm Verification Techniques. *IEEE Transactions on Software Engineering*, 37(6):845–857,2011. 10.1109/TSE.2010.110.

[19] G. Holzmann, D. Peled, and M. Yannakakis. On Nested Depth First Search. In *The Spin Verification System*, volume 32 of *DIMACS*,pages 23–32, 1996. 10.1090/dimacs/032/03.

[20] B. Jacobs, J. Smans, P. Philippaerts, F. Vogels, W. Penninckx, and F. Piessens. VeriFast: A powerful, sound, predictable, fast verifier for C and Java. In *NFM*, 2011. 10.1007/978-3-642-20398-5_4.

[21] B. Jacobs, J. Smans, and F. Piessens. VeriFast: Imperative Programs as Proofs. In *VS-Tools workshop at VSTTE*, 2010.

[22] S. Joosten, W. Oortwijn, M. Safari, and M. Huisman. An Exercise in Verifying Sequential Programs with VerCors. In *FTfJP*, pages 40–45, 2018. 10.1145/3236454.3236479.

[23] G. Kant, A. Laarman, J. Meijer, J. van de Pol, S. Blom, and T. van Dijk. LTSmin: High-Performance Language-Independent Model Checking. In *TACAS*, pages 692–707. Springer, 2015. 10.1007/978-3-662-46681-0_61.

[24] J. Kübler. Comparing Deductive Program Verification of Graph Data-Structures. *Bachelor’s thesis, KIT*, 2018.

[25] A. Laarman, R. Langerak, J. van de Pol, M. Weber, and A. Wijs. Multi-core Nested Depth-First Search. In *ATVA*, LNCS, pages 321–335. Springer, 2011. 10.1007/978-3-642-24372-1_23.

[26] A. Laarman, M. Olesen, A. Dalsgaard, K. Larsen, and J. van de Pol. Multi-core Emptiness Checking of Timed Büchi Automata Using Inclusion Abstraction. In *CAV*, pages 968–983. Springer, 2013. 10.1007/978-3-642-39799-8_69.

[27] P. Lammich and R. Neumann. A Framework for Verifying Depth-First Search Algorithms. In *CPP*, pages 137–146. ACM, 2015. 10.1145/2676724.2693165.

[28] P. Lammich and S. Wimmer. IMP2 – Simple Program Verification in Isabelle/HOL. *Archive of Formal Proofs*, 2019. http://isa-afp.org/entries/IMP2.html, Formal proof development.

[29] K.R.M. Leino. Data groups: Specifying the modification of extended state. In *OOPSLA*, pages 144–153. ACM, 1998. 10.1145/286942.286953.

[30] K.R.M. Leino. Dafny: An Automatic Program Verifier for Functional Correctness. In *LPAR*, pages 348–370. Springer, 2010. 10.1007/978-3-642-17511-4_20.

[31] L. de Moura and N. Bjørner.Z3: An Efficient SMT Solver. In *TACAS*, pages 337–340, 2008. 10.1007/978-3-540-78800-3_24.

[32] P. Müller, M. Schwerhoff, and A. Summers. Viper: A Verification Infrastructure for Permission-Based Reasoning. In *VMCAI*, pages 41–62. Springer, 2016. 10.1007/978-3-662-49122-5_2.

[33] K. Namjoshi. Certifying Model Checkers. In *CAV*, LNCS, pages 2–13. Springer, 2001. 10.1007/3-540-44585-4_2.

[34] P. O’Hearn. Resources, Concurrency and Local Reasoning. *Theoretical Computer Science*, 375(1–3):271–307, 2007. 10.1016/j.tcs.2006.12.035.

[35] W. Oortwijn, M. Huisman, S. Joosten, and J. van de Pol. Artifact for Automated Verification of Parallel Nested DFS, TACAS2020.4TU.ResearchData. 10.4121/uuid:36c00955-5574-44d9-9b26-340f7a1ea03b.

[36] A. Pnueli. The Temporal Logic of Programs. In *FOCS*, pages 46–57. IEEE Computer Society, 1977. 10.1109/SFCS.1977.32.

[37] J. van de Pol. Automated Verification of Nested DFS. In *FMICS*, LNCS, pages 181–197. Springer, 2015. 10.1007/978-3-319-19458-5_12.

[38] A. Raad, A. Hobor, J. Villard, and P. Gardner. Verifying Concurrent Graph Algorithms. In *Programming Languages and Systems*, pages 314–334. Springer,2016. 10.1007/978-3-319-47958-3_17.

[39] J. Reif. Depth-First Search is Inherently Sequential. *Information Processing Letters*, 20(5):229–234, 1985. 10.1016/0020-0190(85)90024-9.

[40] E. Renault, A. Duret-Lutz, F. Kordon, and D. Poitrenaud. Variations on Parallel Explicit Emptiness Checks for Generalized Büchi Automata. *STTT*, 19(6):653–673, 2017. 10.1007/s10009-016-0422-5.

[41] S. Schwoon and J. Esparza. A Note on On-the-Fly Verification Algorithms. In *TACAS*, LNCS, pages 174–190. Springer, 2005. 10.1007/978-3-540-31980-1_12.

[42] I. Sergey, A. Nanevski, and A. Banerjee. Mechanized Verification of Fine-Grained Concurrent Programs. In *PLDI*, pages 77–87. ACM, 2015. 10.1145/2813885.2737964.

[43] C. Sprenger. A Verified Model Checker for the Modal $$\rm \mu $$-calculusin Coq. In *TACAS*, LNCS, pages 167–183. Springer, 1998. 10.1007/bfb0054171.

[44] V. Vafeiadis. Concurrent Separation Logic and Operational Semantics. In *MFPS*, ENTCS, pages 335–351, 2011. 10.1016/j.entcs.2011.09.029.

[45] M. Vardi and P. Wolper. Automata-Theoretic Techniques for Modal Logics of Programs. *Journal of Computer and System Sciences*, 32(2):183–221, 1986. 10.1016/0022-0000(86)90026-7.

[46] Why3 gallery of formally verified programs. http://toccata.lri.fr/gallery/graph.en.html*(accessed on February 2020)*.

[47] S. Wimmer and P. Lammich. Verified Model Checking of Timed Automata. In *TACAS*, LNCS, pages 61–78. Springer, 2018. 10.1007/978-3-319-89960-2_4.

